# Augmenting self-guided virtual-reality exposure therapy for social anxiety with biofeedback: a randomised controlled trial

**DOI:** 10.3389/fpsyt.2024.1467141

**Published:** 2024-11-12

**Authors:** Preethi Premkumar, Nadja Heym, James A. C. Myers, Phoebe Formby, Steven Battersby, Alexander Luke Sumich, David Joseph Brown

**Affiliations:** ^1^ Division of Psychology, London South Bank University, London, United Kingdom; ^2^ Department of Psychology, Nottingham Trent University, Nottingham, United Kingdom; ^3^ Department of Psychology, University of Sheffield, Sheffield, United Kingdom; ^4^ Independent Researcher, Nottingham, United Kingdom; ^5^ Department of Computer Science, Nottingham Trent University, Nottingham, United Kingdom

**Keywords:** social anxiety, longitudinal, perceived control, physiological arousal, presence

## Abstract

**Introduction:**

We previously found that self-guided Virtual Reality Exposure Therapy (VRET) improved Public Speaking Anxiety (PSA) and reduced heartrate. Elevated heartrate characterises social anxiety and the self-guided VRET seemed to reduce heartrate. Thus, receiving continuous biofeedback about physiological arousal during the VRET could help socially anxious individuals to manage their anxiety. The present study aimed to determine whether biofeedback enhances the responsiveness of VRET.

**Methods:**

Seventy-two individuals with high self-reported social anxiety were randomly allocated to VRET-plus-biofeedback (n=38; 25 completers) or VRET alone (n=35; 25 completers). Three hour-long VRET sessions were delivered over three consecutive weeks. During each session, participants delivered a 20-minute public speech in front of a virtual audience. Participants in the VRET-plus-biofeedback group received biofeedback on heartrate and frontal alpha asymmetry (FAA) within the virtual environment and were asked to lower their arousal accordingly. Participants in both groups completed psychometric assessments of social anxiety after each session and at one-month follow-up.

**Results:**

PSA improved by the end of treatment and overall social anxiety improved one month after the VRET across both groups. The VRET-plus-biofeedback group showed a steadier reduction in FAA in the first VRET session and a greater reduction in self-reported arousal across the three sessions than the VRET-alone group.

**Conclusion:**

Biofeedback can steady physiological arousal and lower perceived arousal during exposure. The benefits of self-guided VRET for social anxiety are sustained one month after therapy.

## 1 Introduction

Social Anxiety Disorder (SAD) is a marked fear of social situations especially when it involves scrutiny by others (1). People with SAD fear being observed (e.g., eating or drinking), interacting with others and performing before an audience (e.g., public speaking), and they may avoid these situations altogether (1). SAD is the third most reported psychiatric disorder after depression and alcoholism (2), with a lifetime prevalence of 4% worldwide (3). One in three young people now meet the criteria for SAD globally, while one in six deny having social anxiety (4). In the U.K., 0.6% (n=63 of 10,108 residents) were diagnosed with SAD and SAD was most often comorbid with depression (5). While this proportion is low, it is underdiagnosed (4, 6) and undertreated (7, 8). Thus, SAD poses a significant public health risk.

### 1.1 Self-guided virtual-reality therapy for social anxiety

Recent advances in virtual-reality (VR) technology have resulted in VR-based psychological therapy where realistic scenarios elicited similar emotional responses to that of *in vivo* situations (9, 10). VRET is especially effective for anxiety because clients can encounter anxiety-provoking cues in a controlled manner (11–13). Consequently, VR exposure therapy (VRET) has been found to be as effective as *in vivo* exposure therapy for SAD (14). Patients even prefer VRET over *in vivo* exposure therapy (15, 16).

A further development in VRET is the switch from therapist-led to self-guided intervention (17). Self-guided VRET circumvents the involvement of a therapist, as a non-specialist practitioner can oversee the users’ adherence to the digital intervention (11, 18–23). Thus, the benefits of self-guided VRET are that it has a minimal need for a trained therapist (24, 25) and it has reduced economic costs. Self-guided VRET produces notable decreases in self-reported anxiety among those with panic disorder (22) and specific phobias (19, 26). Furthermore, self-guided VRET is already adopted by the National Health Services in England to reduce agoraphobia (25). When considering SAD, self-guided VRET reduced social anxiety more than waitlist after four sessions, with improvement being sustained for six months after exposure (23). In another study (21), the improvement in Public Speaking Anxiety (PSA) following a session of self-guided VRET was comparable to that of a session of therapist-led VRET and this improvement was sustained for 6-12 months after exposure. However, a single session of VRET (21) may not be reliable. A more involved three-week self-guided VRET for PSA was delivered to adolescents at home (20). Here, adolescents could engage in 15 short (two-minute) public-speaking tasks amounting to 60 minutes of VRET each week. The VRET improved PSA more than being on a waitlist. However, this improvement relied on self-report, rather than objective measures of PSA. Thus, further research is needed to objectively test the benefits of several sessions of self-guided VRET for social anxiety.

### 1.2 Avoidance behaviour and perceived control

Avoidance behaviour is a hallmark of anxiety disorder, where the intolerance to uncertainty generates excessive anxiety and perpetuates avoidance behaviour (27). Anxiety is associated with a reduced sense of control (28), but importantly, the avoidance behaviour itself can facilitate a sense of perceived control over the uncertainty of events, which will then further reinforce avoidance behaviour to maintain control (29). Thus, the more anxious individuals are the less control they perceive and the more they are motivated to avoid the situation. However, ‘perceived’ control could also be facilitated by the amount of control one has over the exposure to a specific threat when it must be approached, such as control over the perceived distance from threat (11). As such, increasing a sense of control over the gradual exposure to threat in self-guided VRET may help reduce uncertainty and avoidance when one must approach an unknown/risky environment and facilitate engagement and exploration of the virtual environment. Indeed, greater perceived control over exposure to fearful stimuli relates to greater willingness to approach threat (30). According to the Health Belief Model (HBM), individuals engage better with treatment when they believe they have fewer barriers to action (31, 32) and possess control over the therapy (31, 32). Thus, socially anxious people are more willing to engage in performing and even give better performances when they have greater perceived control (33).

Having a sense of presence in the virtual environment could facilitate such perceived control and it is another mechanism of improving the efficacy of VRET. Evidence suggests that having a sense of presence in the virtual environment determines the level of improvement in anxiety in both therapist-led VRET and self-guided VRET (18, 34). These studies of self-guided VRET for social anxiety (11, 20, 21, 23) did not examine role of perceived control or sense of presence as of improvement in social anxiety.

### 1.3 Using biofeedback to measure treat anxiety

Elevated physiological arousal, such as increased heartrate during an oral presentation (35, 36), is a hallmark of social anxiety. A month of therapist-led VRET for PSA reduced elevated heartrate (37). Likewise, Premkumar and colleagues (2021) found a reduction in heartrate that co-occurred with a reduction in self-reported social anxiety and PSA over two sessions of self-guided VRET. Heartrate can also be measured as variability (HRV), the variation in beat-to-beat heartrate intervals (38). Here, elevated HRV is linked to an adaptive and healthy cardiovascular system (39). Reduced HRV indicates a maladaptive autonomic nervous system (40–44) and is associated with greater psychological distress and fear and avoidance of social interaction (45).

Frontal alpha asymmetry (FAA), especially rightward (right > left hemisphere activity) asymmetry, represents another biomarker of avoidance behaviour. Conversely, leftward FAA indicates an inclination to approach threat (46) and, in the context of social anxiety, it could denote a willingness for social interaction. Accordingly, highly socially withdrawn individuals have greater rightward FAA when preparing for a speech than less socially withdrawn individuals (47). However, groups high and low in social anxiety did not differ in FAA before and after delivering a speech (48) which could imply that heightened FAA may only relate to social withdrawal and not social anxiety.

Biofeedback about such physiological arousal is important for treating anxiety disorders (49–52) because participants can see the real-time feedback of their physiological reactions and alter their arousal (53). Biofeedback improves the sensation and interpretation of internal physiological signals (54, 55), and aids the practice of emotion regulation (56). Several meta-analyses have noted that biofeedback based on HRV is associated with lower self-reported stress and anxiety (54, 56–58). Biofeedback could even enhance perceived control since learning to synchronise respiration rate with observed heartrate and to relax increases perceived control over situations (55, 59) and enhances creativity (60). In turn, greater perceived control through effective emotional regulation lowers heartrate (61). Thus, therapies use biofeedback to manage anxiety (58) in both clinical (50, 62–65) and non-clinical populations (51, 52, 66). A meta-analysis of randomised controlled trials of biofeedback for anxiety disorders revealed greater improvement in anxiety in the biofeedback-based intervention (broadly defined) than waitlist, but weaker improvement than an active control (67), thus implying the modest benefits of biofeedback as a standalone intervention. If VRET reduces physiological arousal and distress, giving continuous biofeedback to participants about their physiological arousal could help socially anxious individuals to manage their distress. When VR therapy for anxiety includes biofeedback, five out of seven studies reported significant, albeit modest, reductions in self-reported anxiety (Hedge’s g=0.28) and heartrate (g=-0.45) (53). However, these studies mostly delivered a single session of treatment (53).

### 1.4 Aims and hypotheses

The current study aimed to evaluate the benefit of biofeedback on heartrate, FAA and responsiveness to self-guided VRET in social anxiety (11, 68). It was hypothesised that compared to self-guided VRET alone, improvements will be greater for self-guided VRET+biofeedback in terms of:

1. PSA, social anxiety and confidence as a speaker,
2. Continuous self-assessment of anxiety and arousal during VRET sessions, and
3. Physiological arousal (heartrate and leftward FAA).
  In addition, it was hypothesised that across both groups
4. Improvement in social anxiety would be sustained for one month,
5. Perceived control would explain the long-term improvement in PSA and social anxiety, and
6. A greater sense of presence in the virtual environment would predict greater responsiveness to treatment.

## 2 Methods

### 2.1 Participants

Six-hundred and sixty-five participants from the general population completed the initial screening survey. Participants were recruited by placing posters around the university, in local general medical practices, libraries and community centres and on social networking platforms, such as Twitter and Facebook. Three hundred and ninety-seven participants (60%) scored 32 and above on the Social Phobia Inventory (SPIN), indicating moderate-to-high social anxiety (69). Participants were invited to the randomized controlled trial (RCT), the next stage of the study, if they scored >19 on the SPIN, a score which has 79% accuracy with detecting social anxiety (69). Participants were recruited for the RCT until a target of n=75 was reached, namely VRET+Biofeedback, n=38 and VRET alone n=37. Other barriers to participation might have led to participants self-selecting for the RCT, such as motivation to travel to attend an in-person session, meet strangers and confront anxiety in the intervention. Seventy-three participants were recruited and successfully completed the first session. SPIN scores ranged from 20 to 67 (mean=46 ± 10) in the final sample (n=73), suggesting high levels of social anxiety. Participants mostly represented those from the East Midlands region of England who were aged 18 years and above, and had normal or corrected vision with contact lenses as they needed to see the virtual environment clearly. Participants were randomized to VRET+biofeedback and VRET-alone groups and age, sex and ethnicity were similarly distributed between the groups. Likewise, the groups were matched in the level of social anxiety and the number diagnosed with social anxiety disorder or other psychiatric disorder (**Table 1**).

**Table 1:** Comparison between VRET+biofeedback and VRET alone groups on demographic characteristics and change in anxiety over the course of the three sessions of the VRET and one-month follow-up.

Outcome measure	VRET+Biofeedback (n=38)	VRET alone (n=35)	Cohen’s d	Chi-square or F-value (*p*)
*Age	25.47 (9.72)	30.44 (12.34)	0.45	3.54 (0.065)
Sex (% female)	86.8	62.9		5.97 (0.051)
Ethnicity (% White)	76.3	57.1		3.03 (0.081)
Social anxiety disorder diagnosis (% with current or past diagnosis)	21.1	28.6		0.55 (0.457)
Other psychiatric diagnosis (% with current or past diagnosis)	48.0	29.2		1.83 (0.176)
SPIN at baseline	46.4 (10.89)	45.71 (9.72)	0.68	0.08 (0.772)

*Welch test was performed due to significant heterogeneity of variance.

### 2.2 Materials

#### 2.2.1 Social Phobia inventory

The SPIN (69) was used to screen for social anxiety. The 17 items assess self-reported fear, avoidance and physical sensations associated with social anxiety. Items were rated on a five-point Likert scale from 0 = “Not at all” to 4 = “Extremely”. Scores range from 0 to 68, and individuals who meet the DSM-IV diagnostic criteria for social anxiety typically have a mean score>40 (70). The SPIN has adequate to good internal consistency (Cronbach’s alpha >0.80 in 69; 0.94 in the current study), test-retest reliability (r=0.78 and 0.89) and convergent validity (69).

#### 2.2.2 Personal Report of Confidence as a Speaker

The short form of the PRCS scale (71) was used to measure PSA. It consists of 12 true-or-false items on fear of public-speaking. Its psychometric properties include convergent validity with measures of social anxiety and divergent validity with a measure of sociability (71, 72). The PRCS has good internal reliability (Cronbach’s alpha=0.85, 72) (**Table 2**). The summary score was the average rating of all the items.

**Table 2:** Comparison between completers and non-completers of the three VRET sessions on the outcome measures at baseline.

Outcome measure	Cronbach’s alpha (n=73)	Completers Mean (SD)	Non-completers Mean (SD)	Cohen’s d	*F* (df)	*p* value	Group-by-Completion status interaction *F* (df)^†^	*p* value
Baseline								
		n=50	n=23					
PRCS	0.85	0.73 (0.19)	0.68 (0.26)	0.24	0.87 (1,71)	0.353	0.92 (3,69)	0.435
Avoid giving a presentation	NA	78.20 (16.97)	74.39 (25.78)	0.19	0.56 (1,71)	0.455	1.26 (3,69)	0.295
PSA	0.94	65.12 (9.93)	62.56 (9.98)	0.26	1.04 (1,71)	0.311	0.48 (3,69)	0.697
BFNE	0.88	35.44 (7.53)	33.48 (10.49)	0.23	0.83 (1,71)	0.366	0.54 (3,69)	0.659
LSAS – Fear of Performance	0.85	20.02 (7.99)	19.39 (10.29)	0.07	0.08 (1,71)	0.777	0.69 (3,69)	0.652
LSAS – Fear of social situations	0.91	18.72 (8.27)	17.74 (9.70)	0.11	0.20 (1,71)	0.657	0.84 (3,69)	0.475
^‡^LSAS – Avoidance of Performance	0.82	17.77 (7.45)	17.00 (8.29)	0.10	0.15 (1,71)	0.698	0.77 (3,66)	0.517
^‡^LSAS – Avoidance of social situations	0.88	17.45 (8.14)	16.30 (8.29)	0.14	0.30 (1,71)	0.585	0.86 (3,66)	0.464
RST – BIS appraisal	0.82	3.29 (0.69)	3.26 (0.75)		0.01 (1,71)	0.885	1.27 (3,66)	0.292
Post-session 1								
		n=48	n=15					
Presence – realism	0.82	34.18 (6.73)	31.20 (7.67)	0.43	2.11 (1,61)	0.152	0.63 (1,58)	0.432
Presence – possibility to act	0.63	20.42 (3.56)	19.47 (4.60)	0.25	0.70 (1,61)	0.405	2.20 (1,58)	0.143
Presence – quality of interface	0.66	12.17 (5.15)	9.80 (3.86)	0.48	2.68 (1,61)	0.107	2.46 (1,58)	0.122
Presence – possibility to examine	0.71	14.29 (3.41)	13.27 (4.06)	0.29	0.94 (1,61)	0.335	0.71 (1,58)	0.402
Presence – self-evaluation of performance	0.76	11.12 (2.01)	9.47 (2.12)	0.81	**7.46 (1,61)**	**0.008**	0.50 (1,58)	0.484

^†^Completers and non-completers in the VRET+biofeedback group = 25 and 13; completers and non-completers in the VRET-alone group = 25 and 10; ^‡^Completers and non-completers in the VRET+biofeedback group = 22 and13; completers and non-completers in the VRET-alone group = 25 and 10; BFNE, Brief Fear of Negative Evaluation scale; LSAS, Liebowitz Social Anxiety Scale; NA, Not applicable because the scale is a single item; PRCS, Personal Report of Confidence as a Speaker scale; PSA, Public-Speaking Anxiety scale; RST_BIS, Reinforcement Sensitivity Theory – Behavioural Inhibition Scale; RST_BAS, Reinforcement Sensitivity Theory – Behavioural Approach Scale. Values in bold are statistically significant.

#### 2.2.3 Public-Speaking Anxiety

The PSA scale (73) is a 17-item measure of cognitive, behavioural and physiological dimensions of PSA. Items were rated on a five-point Likert scale from 0 = “Not at all” to 4 = “Extremely”. The sum of individual items was calculated after reverse scoring some items. Scores range from 17 to 85. The scale’s significant correlations with other self-reported measures of speech anxiety and measures of anxiety, but weak correlation with a measure of depression evidence the scale’s good concurrent validity, convergent validity and discriminant validity, respectively (73). The internal consistency was good in the original development study (Cronbach’s alpha=0.94; 73) and the current study (**Table 2**).

#### 2.2.4 Liebowitz Social Anxiety Scale

The LSAS (74) measures fear and avoidance in different social and performance situations. Twelve statements concern social interactions, such as going to a party and meeting strangers. A further 12 items concern performance situations, such as eating in public spaces and working under observation. For each statement, participants were asked how much they feared that situation and how much they avoided the situations. The Likert scale ratings of fear were 0 = “None”, 1 = “Mild”, 2 = “Moderate” and 3 = “Severe”. The Likert scale ratings of avoidance were 0 = “Never (0%)”, 1 = “Occasionally (1—33%)”, 2 = “Often (33—67%)” and 3 = “Usually (67—100%)”. Thus, there are four subscales, Fear of Performance situations, Avoidance of Performance situations, Fear of Social interaction situations and Avoidance of Social interaction situations. The summary score of each subscale was the sum of all the items in that subscale. The scale has good convergent validity with other measures of social anxiety (75). The four subscales had acceptable to good internal consistency in the current study (**Table 2**).

#### 2.2.5 Brief Fear of Negative Evaluation

The BFNE scale (76) has 12 items on the fear of being evaluated by others. FNE is where the person is concerned that others will think badly of them and criticise them (77). FNE is a hallmark of PSA and social anxiety (77, 78). The items were rated on a five-point Likert scale from 0 = “Not at all characteristic of me” to 0 = “Extremely characteristic of me”. The summary score was the sum of all the items after reverse scoring some items. The scale demonstrated good internal reliability in previous studies (76, 79, 80) and the current study (**Table 2**), and good test-retest reliability (76, 81). The BFNE has good construct validity since patients with social anxiety score higher than non-anxious patients on the BFNE (80).

#### 2.2.6 Visual analogue scale ratings of behavioural avoidance, arousal and anxiety

Participants rated VASs from 0 to 100 within the virtual environment at each session (a) before entering the virtual lecture room, (b) during each pause and (c) after leaving virtual lecture room. The VASs measured avoidance of giving a speech (this alone was assessed before and after each VRET session), arousal and anxiety. Arousal was defined as feeling vigorous, lively, energetic and alert. Anxiety was defined as dryness of mouth, difficulty breathing, trembling, feeling panicked, increased heartrate and scared.

#### 2.2.7 Presence questionnaire

Nineteen items measure sense of presence in the virtual environment (82). Items were rated on a seven-point Likert scale with the descriptors of the anchor points varying from item to item. The scale has five subscales with poor to good internal reliability (**Table 2**). *Realism* refers to how natural and compelling the environment was. *Possibility to Act* enquires about controlling and surveying the environment. *Quality of Interface* gauges delays to one’s actions appearing in the environment and being distracted by the environment when completing the task. *Possibility to Examine* refers to examining the environment closely. *Self-evaluation of Performance* involves adjusting to and being proficient with interacting with the environment.

#### 2.2.8 Behavioural Inhibition System – appraisal subscale

The Behavioural Inhibition System (BIS)-appraisal subscale of an inhouse measure of reinforcement sensitivity (83) was used as a proxy measure of perceived control. BIS relates to perceived control over negative life events (28). Furthermore, locus of control partly explains the relationship between BIS and trait anxiety (84). BIS-appraisal is seen as an essential component of anxiety that involves monitoring risk and carefully appraising information about uncertainty, weighing up the pros and cons of a situation before engaging in approach or avoidance behaviour (27). This in-house BIS-appraisal subscale forms part of a measure of trait anxiety and consists of four items that are rated on a four-point Likert scale from 1 = “Very false for me” to 4 = “Very true for me”.

### 2.3 Physiological arousal measures

#### 2.3.1 Heartrate

**Figure 1:**
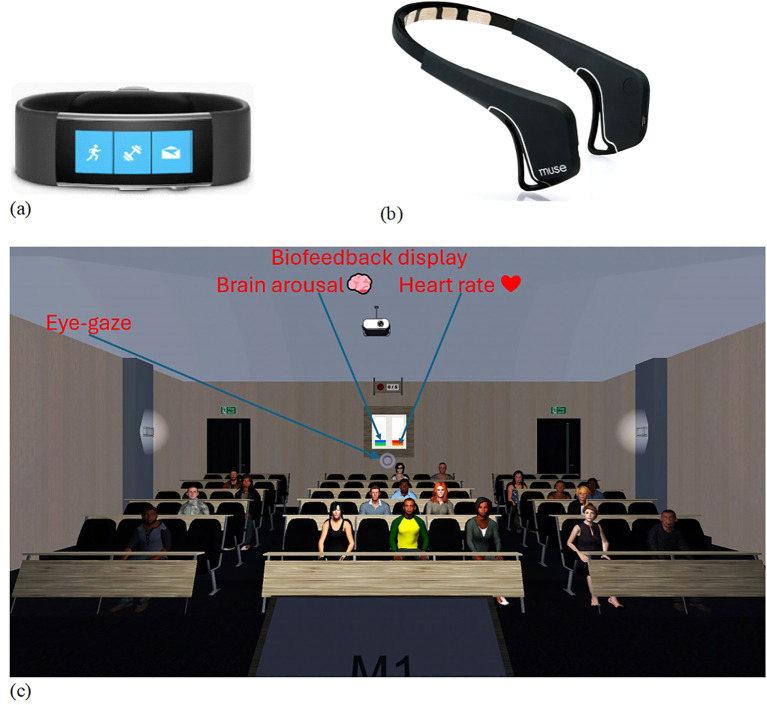
Physiological arousal measurement using **(A)** Microsoft Band 2 biometric wrist band to measure heart rate and **(B)** Muse brain sensing headband to measure frontal alpha asymmetry, and **(C)** virtual-reality lecture hall with biofeedback display.

Heartrate was measured continuously during the public speech in each VRET session. Data were collected from a Microsoft Band 2 biometric wristband which has 11 sensors for tracking heartrate and blood pressure (**Figure 1A**). Heartrate was sampled at 10 Hz and the average beats per minute were calculated for each four-minute speech block during the VRET session.

#### 2.3.2 Electroencephalography

Frontal electrical signals were recorded continuously from a Muse wireless EEG headband (**Figure 1B**) (85). Frontal alpha power was sampled at 220 Hz from the AF7 and TP9 channels on the left and AF8 and TP10 channels on the right with the FPz site as the reference. Average frontal alpha power was calculated from the 60 samples per minute during each four-minute speech block. FAA was calculated as (the average of AF7 and TP9 on the left) minus (the average of AF8 and TP10 on the right).

#### 2.3.3 Self-guided VRET software and hardware

A Samsung Gear VR headset housed a Samsung Galaxy S7 smartphone through which the VRET application displayed the virtual environment. The VRET application was developed using the Unity real-time 3D development platform (86). The Unity-based VRET smartphone application was deployed to the Android operating system on the Samsung Galaxy S7 smartphone. Heartrate data were collected through the smartphone application, which was connected to Microsoft Band 2. A bespoke plugin developed in Java bridged the Java-based official Microsoft Band software to the VRET smartphone application.

### 2.4 Virtual environment design and self-guided manipulation

Participants gave a 20-minute speech about “going on a holiday”. The session was broken into five four-minute speech blocks. Participants spoke spontaneously using prompts (e.g., dream destination and sight-seeing) that appeared on a podium in the virtual environment. After each speech block, participants had a brief (one minute) pause to perform the following tasks, namely respond to VASs on anxiety and arousal, navigate to a ‘settings menu’ and change the exposure level of five exposure elements. Each modifiable element had three grades (G) of exposure ranging from low to high: (i) audience size consisting of 6 (G1), 12 (G2) or 20 people (G3); (ii) audience reaction comprising approving (G1), neutral (G2) or disapproving reactions (G3); (iii) speaker’s distance from the audience being far (G1), near (G2) or nearest (G3); (iv) number of speech prompts per slide, each slide having many (G1), moderate (G2) or few prompts (G3); and (v) salience of self having no poster (G1), a silhouette with the label “Speaker” (G2), or a photo of the participant and their full name (G3). Participants were encouraged to increase their exposure to threat at their own pace (see 11, for further details). Those in the VRET+biofeedback group received continuous biofeedback about their arousal level and were asked to monitor and lower their arousal accordingly. The biofeedback consisted of two vertical bars displayed on the rear of the virtual lecture theatre that oscillated, with the red bar denoting heartrate and the blue bar denoting FAA (**Figure 1C**).

### 2.5 Procedure

Ethical approval was obtained from the university’s Research Ethics Committee, ethics application number No. 2017/115. Participants gave informed consent and received a £15 shopping voucher for each VRET session (69). Participants were randomly allocated to the two interventions single-blind using a randomisation list. Before session one, participants completed an online survey containing the BFNE, LSAS, PRCS and PSA scale for the baseline assessment. Participants in the two groups did not differ across those measures at baseline, *F*(1,71)<2.14, *p*>0.148. The PRCS was readministered after each VRET session and at four-week follow-up. The Presence Questionnaire was administered after the first VRET session. The BFNE, LSAS and PSA scale were readministered at the end of therapy (after the three sessions) and at one-month follow-up. The sessions were held in the same laboratory throughout the study. Thus, the ambient room temperature was monitored to ensure that the change in temperature did not alter physiological arousal.

### 2.6 Statistical analysis

#### 2.6.1 Missing data analysis and manipulation checks

Chi-square tests compared the number of completers in each group at each session. Analyses of variance (ANOVAs) compared treatment completers and non-completers on each self-reported scale at baseline. The analyses were repeated with Group as an additional independent variable (IV). These analyses determined whether multiple imputation could replace missing data from non-completers at subsequent sessions. Multiple imputation was then performed using the iterative Markov Chain Monte Carlo method.

#### 2.6.2 Hypothesis-testing

A series of 2x2 mixed-design ANOVAs was performed with group (VRET+biofeedback and VRET-alone) and time (baseline, post-treatment) as the IVs and measures of social anxiety as the dependent variables (hypothesis 1). Two 2x3x4 mixed-design ANOVAs were performed with Group (VRET+biofeedback vs. VRET-alone), Time (sessions 1, 2 and 3) and Block (1, 2, 3 and 4) as IVs and the self-reported VASs of arousal and anxiety as the dependent variables (DVs) (hypothesis 2). Two further 2x2 ANOVAs were performed with Group (VRET+biofeedback vs. VRET-alone) and Time (first minute of the first session and last minute of the third session) as IVs, and heartrate and FAA as the DVs (hypothesis 3). Another ANOVA was performed with Group (VRET+biofeedback vs. VRET-alone), Minute (1, 2, 3 and 4) and Block (1, 2, 3 and 4) as IVs, and heartrate and FAA at just the first VRET session as the DVs (hypothesis 3).

2x3 ANOVAs using quadratic contrasts as the model of comparison were performed with Group (VRET+biofeedback vs. VRET-alone) and Time (baseline, post-treatment and one-month follow-up) as the IVs, and the scores on BFNE, LSAS, PRCS and PSA scale as the DVs to test long-term improvement in social anxiety (hypothesis 4). Then, analyses of covariance (ANCOVAs) were performed on the same measures with the appraisal subscale of the rRST as a covariate (hypothesis 5). Multiple linear regressions were performed with the subscales of the Presence Questionnaire at session one as the predictor variables and change-relative-to-baseline on PSA and social anxiety as the criterion variables (hypothesis 6). Change relative to baseline was calculated as follows,


$$\text{Change relative to baseline}=\frac{\text{Score}\;\text{at}\;\text{baseline}\;\;–\;\text{Score at end of treatment}}{\text{Score at baseline}}\times 100.$$

## 3 Results

### 3.1 Comparison between completers and non-completers on outcome measures

The number of completers in the VRET+biofeedback group was 34 (89%), 29 (76%), 25 (66%) and 19 (45%) at sessions 1, 2 and 3 and one-month follow-up, respectively. The number of completers in the VRET-alone group was 29 (81%), 26 (72%), 25 (69%) and 23 (64%) at sessions 1, 2 and 3 and one-month follow-up respectively. There was no difference between groups in the rate of dropout at any session, Chi-square<1.25, P>0.228. Completers and non-completers did not differ on any social anxiety measure at baseline (**Table 2**). Therefore, missing data of non-completers were replaced with predicted scores obtained from multiple imputation on all social anxiety measures. Completers also rated the ‘self-evaluation of performance’ subscale of the Presence Questionnaire at the end of session 1 higher than the non-completers.

### 3.2 Change in PSA and social anxiety from baseline to end-of-treatment

**Figure 2:**
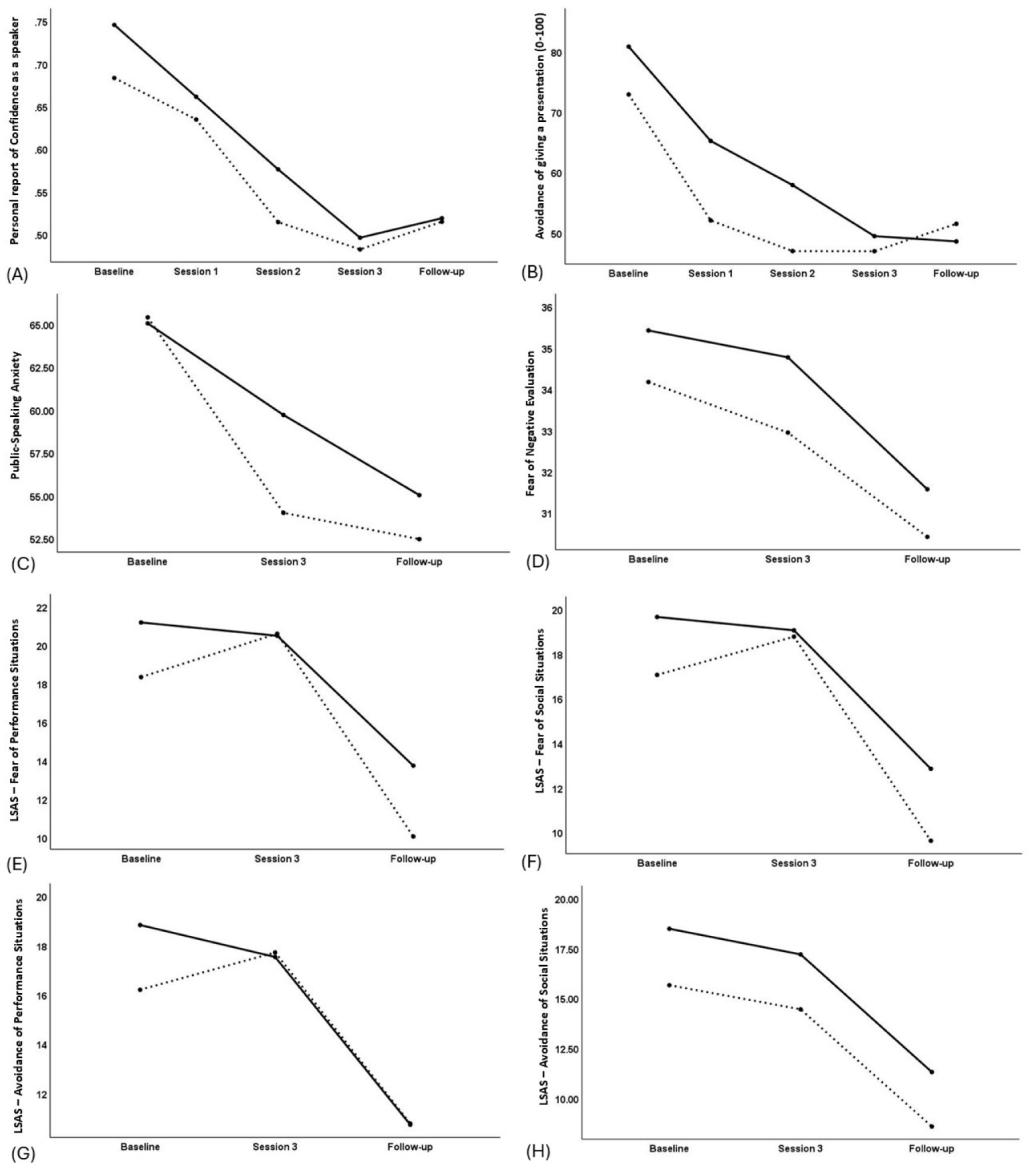
Plot of time (x-axis) by outcome measure (y-axis) in the VRET+biofeedback group (solid line) and VRET-alone group (broken line) for the following outcome measures, **(A)** Personal Report of Confidence as a Speaker, **(B)** Avoidance of giving a presentation, **(C)** Public-speaking Anxiety, **(D)** Brief Fear of Negative Evaluation, **(E)** Liebowitz Social Phobia Scale – Fear of Performance situations, **(F)** Liebowitz Social Anxiety Scale – Fear of Social Situations, **(G)** Liebowitz Social Anxiety Scale – Avoidance of Performance situations, **(H)** Liebowitz Social Anxiety Scale – Avoidance of Social situations. For all scales, greater reduction relative to baseline means greater improvement in anxiety.


There was a main effect of Time on PSA, *F*(1, 71)=42.23, *p*<0.001, PRCS, *F*(1, 71)=53.39, *p*<0.001 and avoidance of giving a speech, *F*(1, 71)=116.24, *p*<0.001, suggesting improvement in PSA by the end of treatment (**Figures 2A-C**). The reliable change index (RCI) (20, 87) was used to determine if the change was clinically meaningful,

$$\text{RCI=}\frac{x_{2}-x_{1}}{\sqrt{2{\left(SE\right)}^{2}}}$$

where x_2_ is the score at end of treatment (or follow-up) and x_1_ is the score at baseline. SE was the standard error of the difference between the two sets of scores. An RCI>1.96 is considered clinically meaningful. The change was clinically meaningful for each measure, RCI_PSA_ = -4.4, RCI_PRCS_ = -5.4, RCI_avoidance_ = -7.6, where a negative sign means a reduction in the scores.

However, the main effect of Time was not significant for BFNE or LSAS, *F*(1,71)<2.66, *p*>0.108 (**Figures 2D-H**). There was a trend for greater improvement in PSA in the VRET-alone group than the VRET+biofeedback group (**Table 3**; **Figure 2C**). Correspondingly, RCI_PSA_=-2.1 in the VRET+biofeedback group and RCI_PSA_=-4.5 in the VRET alone group. There was a trend for greater improvement on LSAS-avoidance of performance in the VRET+biofeedback group than the VRET-alone group (**Figure 2G**). However, these changes did not correspond to clinical significance with clinical significance, RCI_LSPS – avoidance of performance_=-1.4 in the VRET+biofeedback group and RCI_LSPS – avoidance of performance_=-0.9 in the VRET alone group.

**Table 3:** Comparison between VRET+biofeedback and VRET alone groups on demographic characteristics and change in anxiety over the course of the three sessions of the VRET and one-month follow-up.

Outcome measure	VRET+Biofeedback (n=38) Relative change from baseline to follow-up, mean (SD)	VRET alone (n=35) Relative change from baseline to follow-up, mean (SD)	Cohen’s d for group difference in relative change from baseline to follow-up	Change from baseline to end-of-treatment F statistic of Group-by-time interaction (H1)	Change from baseline to follow-up (H2, time points)	*Group-by-time F (H3)	*Change from baseline to follow-up Main effect of time F (*p*)	*Time (P1 to FU) with BIS appraisal as a covariate F (*p*) (H4)
^†^PRCS	25.14 (39.23)	23.8 (34.96)	0.04	0.612 (0.433)	P1, S1, S2, S3, FU	0.04 (0.834)	**10.09 (0.002)**	0.12 (0.731)
^†^Avoidance of giving a presentation	34.20 (37.66)	29.49 (30.09)	0.14	1.039 (0.312)	P1, S1, S2, S3, FU	**4.03 (0.049)**	**37.75 (<0.001)**	0.57 (0.451)
^†^PSA	15.36 (15.75)	17.83 (15.54)	0.16	3.91 (0.052)	P1, S3, FU	**5.83 (0.018)**	**10.58 (0.002)**	0.11 (0.746)
^†^BFNE	8.53 (22.04)	9.06 (24.05)	0.25	0.18 (0.675)	P1, S3, FU	0.28 (0.599)	**22.85 (<0.001)**	0.26 (0.608)
^†^LSAS – Fear of Performance	30.10 (35.79)	51.74 (69.79)	0.40	3.077 (0.084)	P1, S3, FU	**6.22 (0.015)**	**48.88 (<0.001)**	1.84 (0.179)
^†^LSAS – Fear of social situations	22.08 (75.72)	38.68 (40.98)	0.27	2.42 (0.124)	P1, S3, FU	**5.29 (0.024)**	**52.00 (<0.001)**	2.13 (0.149)
^†^LSAS – Avoidance of Performance	42.89 (33.64)	33.36 (40.49)	0.26	3.82 (0.055)	P1, S3, FU	1.62 (0.207)	**36.41 (<0.001)**	2.34 (0.129)
^†^LSAS – Avoidance of social situations	33.48 (38.87)	52.34 (71.30)	0.33	0.002 (0.960)	P1, S3, FU	0.001 (0.979)	**19.49 (<0.001)**	2.47 (0.120)

*F-statistic is based on quadratic contrasts between time points due to the lag in the level of change during the one-month follow-up after the three sessions, unless otherwise specified; ^†^VRET+biofeedback group, n = 38, VRET alone group, n = 35; BFNE, Brief Fear of Negative Evaluation scale; FU, follow-up; LSPS, Liebowitz Social Anxiety Scale; H1, H2 and H3, Hypotheses 1, 2 and 3; P1, baseline, PRCS, Personal Report of Confidence as a Speaker scale; PSA, Public-Speaking Anxiety scale; S1, S2, S3, Sessions 1, 2 and 3, respectively; SPIN, Social Phobia Inventory. Values in bold are statistically significant.

**Figure 3:**
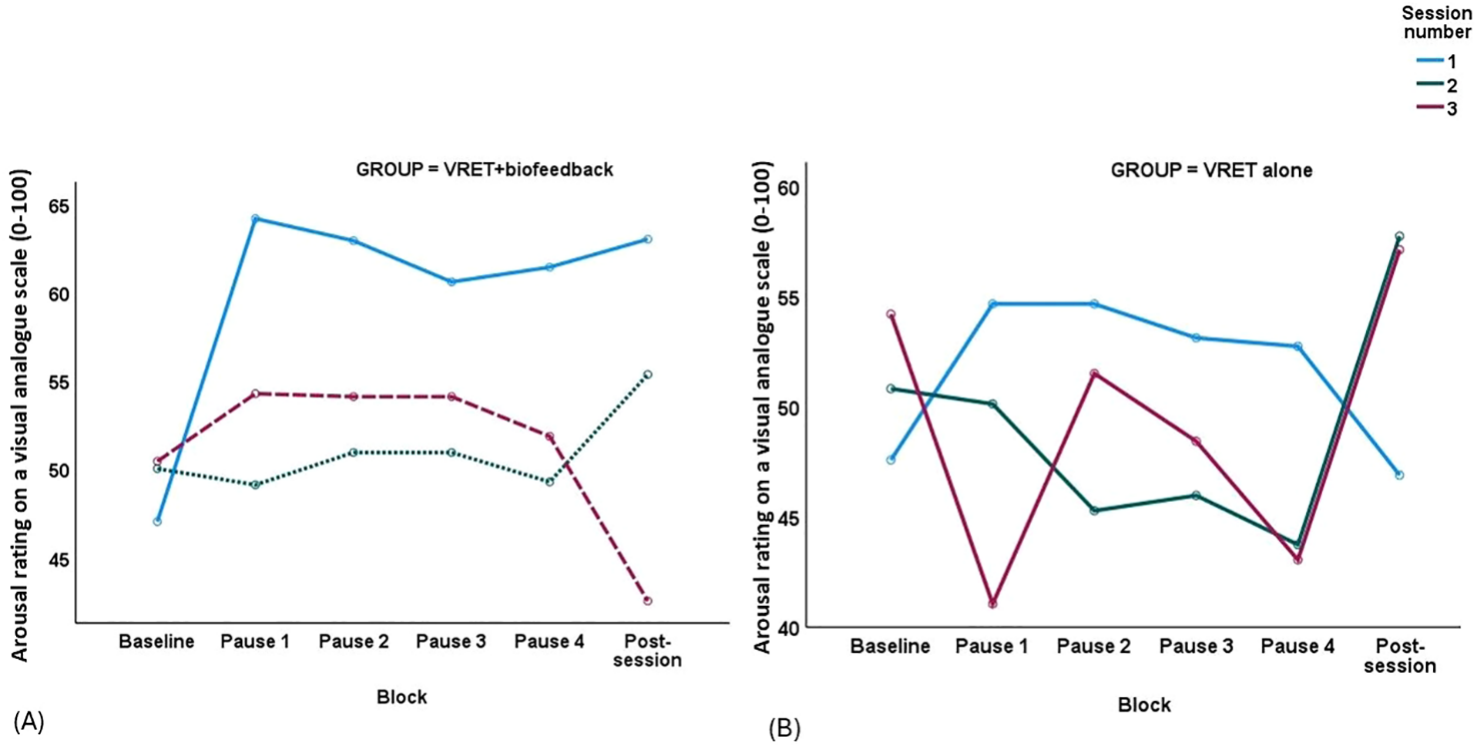
Plot of block (x-axis) by self-reported arousal (y-axis) in **(A)** the VRET+biofeedback group and **(B)** VRET alone group across the three sessions, session 1 – continuous line, session 2 – dotted line and session 3 – broken line.

Some participants in the VRET+biofeedback group (n=12) and the VRET-alone group (n=13) completed the VASs of anxiety and arousal during every pause of all three VRET sessions. There was a marginal gender bias in the likelihood of completing the VASs, with women in the VRET+biofeedback group (n=11) being more likely to complete these VASs than in the VRET-alone group (n=7), χ^2^(1)=3.55, *p*=0.059. There was a Group-by-Session-by-Block interaction for VAS-arousal, *F*(5.6, 130)=2.24, *p*=0.046. According to the plot of VAS arousal (**Figure 3**), self-reported arousal was lower at sessions 2 and 3 of the VRET+biofeedback intervention compared to session 1, and the decline was steadier from one pause to the next in each session. In contrast, self-reported arousal changed haphazardly between sessions and pauses in each session in the VRET-alone group. There was no Group-by-Session-by-Block interaction for VAS-anxiety, *F*(4, 91.8)=0.66, *p*=0.620.

### 3.3 Change in PSA and social anxiety at one-month follow-up

The main effect of Time was significant for all measures of PSA and social anxiety, *F*>10, *p*<0.001 (**Table 3**). Again, these changes were clinically meaningful, RCI_PRCS_ = -5.2, RCI_avoidance_ = -6.3, RCI_PSA_ = -6.3, RCI_BFNE_ = -3.4, RCI_LSPS – Fear of performance_ = -7.3, RCI_LSPS – Fear of social situations_ = -6.7; RCI_LSPS – Avoidance of performance_ = 6.5, RCI_LSPS – Avoidance of social situations_ = -6.4. Furthermore, the Group-by-Time interaction was significant for avoidance of giving a presentation, PSA, LSAS-Fear of Performance and LSAS-Fear of Social Situations (**Table 3**). The effect size (Cohen’s d) of the difference in the improvement between the VRET-alone group and the VRET+biofeedback group was medium for LSAS-Fear of Performance, small for BFNE, LSAS – Fear of social situations, LSAS – Avoidance of Performance and LSAS – Avoidance of social situations and negligible for PRCS, Avoidance of giving a presentation and PSA. Thus, the VRET-alone group showed greater improvement on avoidance of giving a presentation and PSA than the VRET+biofeedback group at the end of treatment, but the improvement levelled between the groups at follow-up (**Figures 2B, C**). The improvement from end-of-treatment to follow-up on LSAS-Fear of Performance and LSAS-Fear of Social Situations was greater in the VRET-alone group than the VRET+biofeedback group (**Figures 2E, F**, respectively).

### 3.4 Improvement social anxiety after covarying for BIS-appraisal at baseline

The main effect of time was no longer significant after covarying for BIS-appraisal for any measure of PSA or social anxiety (**Table 3**).

### 3.5 Change in physiological arousal during the VRET

**Figure 4:**
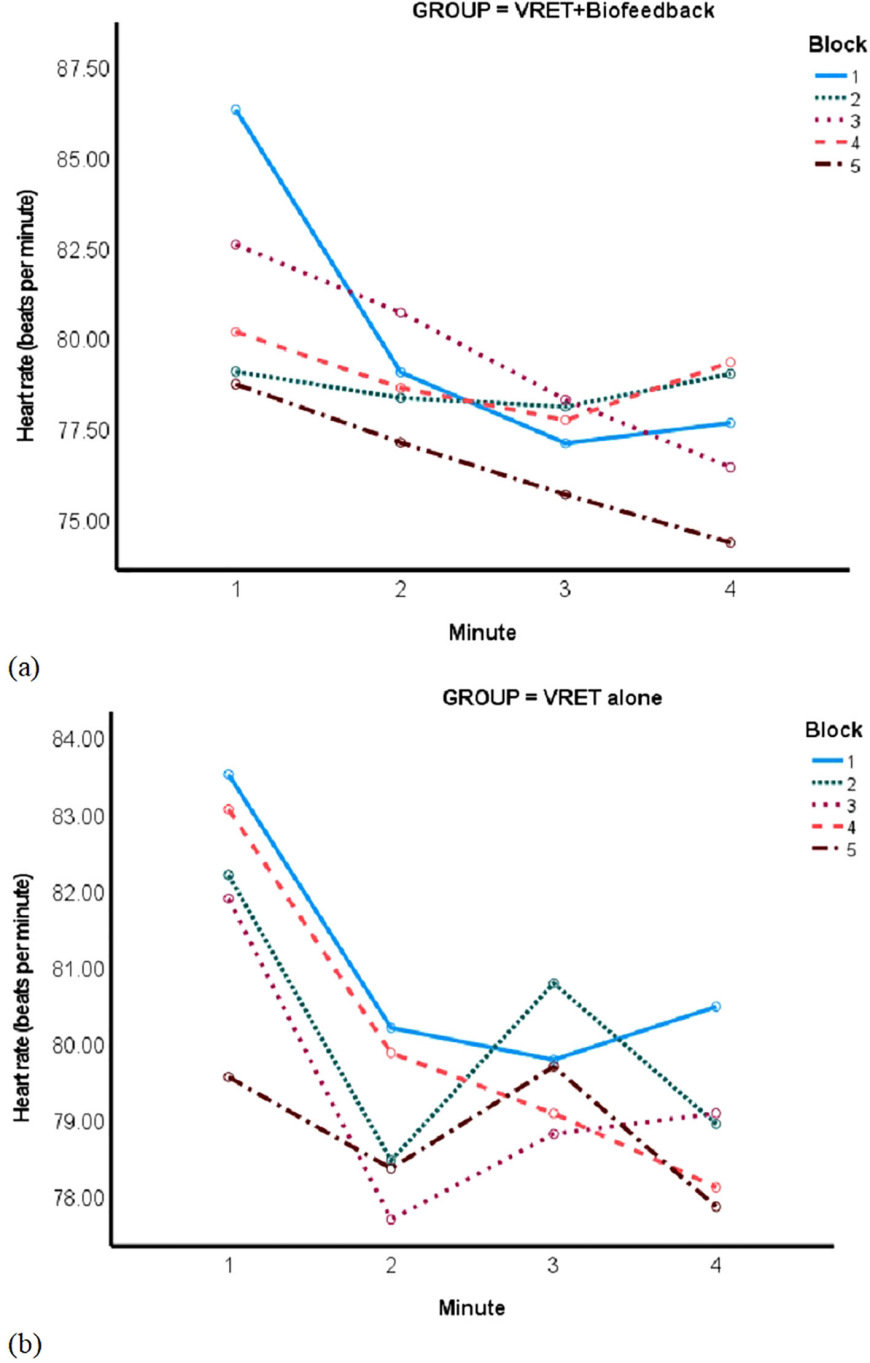
Plot of time in minutes (x-axis) by heartrate (y-axis) by block (separate lines) in **(A)** VRET+biofeedback and **(B)** VRET alone groups.

There was a main effect of time on heartrate among participants with heartrate data at every minute of the four speech blocks and across all three sessions (VRET+biofeedback, n=8, and VRET-alone, n=10), *F*(1, 16)=6.24, *p*=0.024. When examining the change in heartrate at just the first VRET session (VRET+biofeedback, n=22, and VRET-alone, n=16), the Group-by-Block-by-Minute interaction with non-linear (fourth order) contrasts was not significant, *F*(3, 48.3)=0.32, *p*=0.81. Still, a decline in heartrate from one block to the next after the first block appeared steadier the VRET+biofeedback group (**Figures 4A** and **B**).

**Figure 5:**
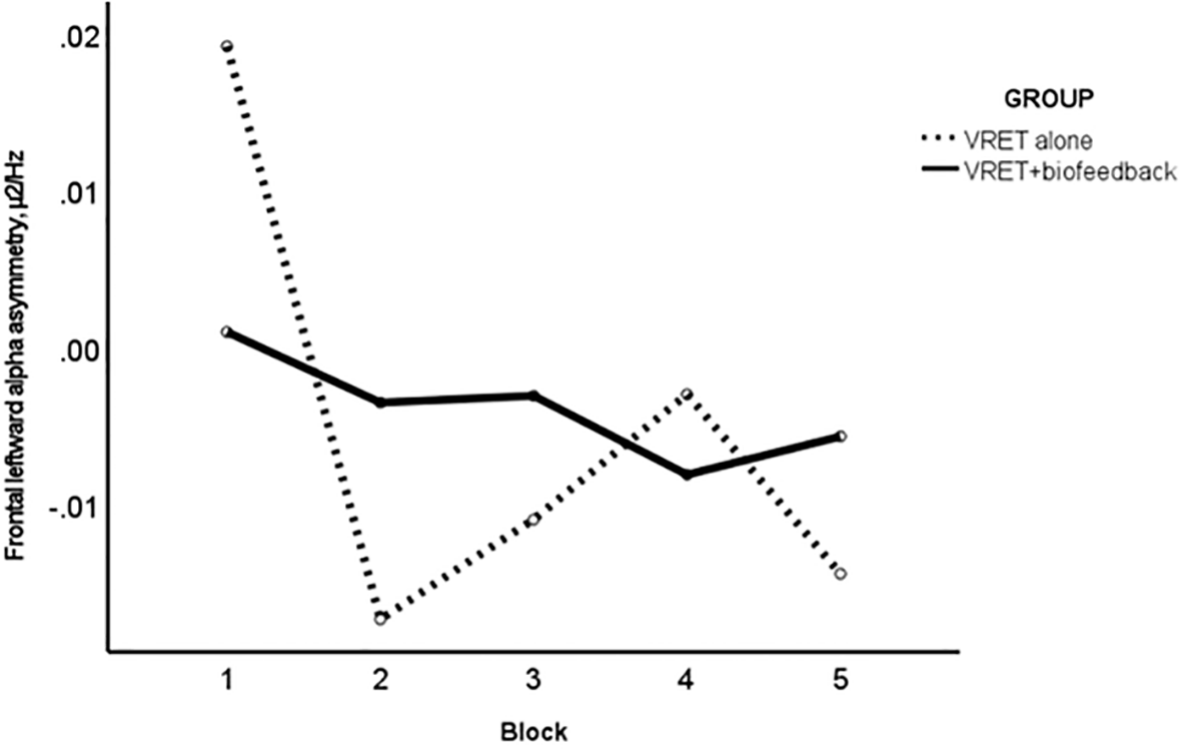
Plot of block (x-axis) by frontal alpha asymmetry (µ^2^/Hz; y-axis) in session 1; VRET+biofeedback (solid line) and VRET alone (broken line).

There was no effect of time on FAA among participants with FAA data at every minute of each VRET session (10 VRET+biofeedback, n=10, and VRET-alone participants, n=12), *F*(1, 20) = 0.33, *p*=0.573. However, the Group-by-Block interaction with non-linear (cubic) contrasts was significant when studying FAA in 20 VRET+biofeedback participants and 16 VRET-alone participants at the first VRET session alone, *F*(1)=5.86, *p*=0.021. The VRET+biofeedback group showed a steady decline in FAA, while the FAA in the VRET-alone group changed haphazardly from one block to the next (**Figure 5**).

### 3.6 Prediction of change in PSA and social anxiety by sense of presence

A greater sense of presence at session 1 predicted improvement in PSA at end of session 3, F (5,55) = 5.58, *p*<0.001 (**Table 4**). Specifically, greater ability to examine significantly predicted improvement in PSA, standardised beta = 0.53, *p*<0.001.

**Table 4:** Multiple regression analysis between subscales of the Presence Questionnaire and change in measures of social anxiety and public-speaking anxiety from baseline to end of therapy (n=62).

	F	R (R^2^)	Standardised Beta				
			Realism	Possibility to act	Quality of interface	Possibility to examine	Self-evaluation of performance
PRCS	1.08	0.30 (0.09)	-0.13	0.14	0.03	0.15	0.16
Avoidance of giving a presentation	2.22	0.41 (0.17)	-0.10	-0.02	-0.01	0.40	0.15
PSA	**5.85**	0.57 (0.35)	-0.22	0.24	-0.13	**0.53**	0.10
BFNE	0.57	0.22 (0.05)	0.16	-0.01	-0.12	0.07	-0.03
LSAS – Fear of Performance	0.44	0.20 (0.04)	0.14	0.14	-0.04	-0.20	-0.07
LSAS – Fear of social situations	0.97	0.29 (0.08)	0.02	0.28	-0.05	-0.08	-0.24
LSAS – Avoidance of Performance	0.42	0.20 (0.04)	0.10	0.05	0.09	-0.12	0.12
LSAS – Avoidance of social situations	0.58	0.23 (0.05)	0.06	0.18	0.09	-0.07	0.03

Values in bold denote correlations that were significant at *p<*0.001.

## 4 Discussion

This is the first RCT to assess the role of biofeedback in the responsiveness of individuals with social anxiety to self-guided VRET. The hypothesis of greater improvement in PSA and social anxiety in the VRET+biofeedback group than the VRET-alone group was not supported (hypothesis 1). However, the hypothesis of greater continuous improvement in self-reported arousal during each VRET session in the VRET+biofeedback group was supported (hypothesis 2). Furthermore, the VRET+biofeedback group showed a steadier reduction in FAA from one block to the next than the VRET-alone group at the first VRET session alone (hypothesis 3). In addition, the hypothesized improvement in PSA and social anxiety one month after therapy in both groups was supported (hypothesis 4). Another hypothesis that perceived control, as measured by BIS-appraisal in this study, would explain the improvement in PSA and social anxiety following self-guided VRET (hypothesis 5) was upheld. Lastly, a greater sense of presence when examining the virtual environment predicted greater improvement in PSA at end of treatment (hypothesis 6).

### 4.1 Improvement at end of treatment and at follow-up

Our findings strengthen the case for self-guided VRET as a potential treatment of choice. Our first study tested university students with high PSA (11). There, a post-treatment improvement was observed in PSA, but not other measures of social anxiety. In the current study too, PSA, but not other measures of social anxiety improved at the end of treatment regardless of the presence or absence of biofeedback. However, the benefits of the self-guided VRET extended to measures of social anxiety at follow-up in the present study, with all measures of PSA and anxiety having a clinically meaningful improvement. The current study tested participants with high social anxiety from the general community and so, this study tested whether the improvement in PSA and social anxiety would generalise to socially anxious individuals. The improvement in PSA at end of treatment was clinically meaningful. This improvement in PSA corresponded with a reduction in heartrate in both groups during each session, suggesting that the improvement in PSA was both perceived and real.

This improvement could be attributed to the sense of presence in the virtual environment in terms of being able to examine elements of the self-guided VRET, such as the five modifiable elements, because it predicted improvement in PSA. A sense of presence within a virtual environment consists of existing in a physical space, in social interaction and experiencing a sense of togetherness with others (88). The association between presence and treatment outcome in the present study may exist because a sense of presence elicits the anxiety that VRET alleviates (89). Sense of presence accounts for improvement in symptoms of acrophobia following self-guided VRET (18). However, sense of presence did not predict treatment outcome following therapist-led VRET for arachnophobia in one study (26), yet it did for agoraphobia in another study (34). Thus, the predictive value of sense of presence may vary by type of phobia, especially when virtual avatars are involved. VRET for SAD is as effective as *in vivo* exposure therapy for SAD (14) and this suggests that patients with SAD can meaningfully perceive threat from a virtual audience and experience a meaningful improvement in FNE. The greater improvement on the BFNE from baseline to follow-up (small effect size) in the VRET-alone group compared with the VRET+biofeedback group suggests that the VRET-alone group may have engaged with the VRET better which may have lasting improvement in how socially anxious individuals perceive the threat of negative evaluation from others. Thus, the threat from a virtual audience could affect how participants interpret others’ evaluation of them even though participants know that the audience is not real.

#### 4.1.1 The role of biofeedback

The VRET+biofeedback group displayed greater improvement in self-reported arousal at sessions 2 and 3 than at session 1, and participants rated their arousal more consistently at each pause than the VRET-alone group. Furthermore, a steadier decline in FAA from the first to the last block of the first VRET session featured in the VRET+biofeedback group. Participants in the VRET+biofeedback group were told to lower the biofeedback bars if they went up. This process of controlling the visual display of physiological arousal may have steadied the participants’ FAA and heartrate and improved their perceived control. The steadier decline in FAA suggests that participants could apply greater cognitive control and gradually lower their arousal. Greater awareness of physiological sensations through biofeedback (54, 55) could improve cognitive control and address the heightened physiological arousal that is a key symptom of anxiety. The findings of the study support the evidence of the benefits of biofeedback as an intervention for anxiety (67) and in combination with VR therapy (53).

Lower heartrate relates to greater perceived control when emotion regulation is high (61). Participants in the VRET+biofeedback group may have learned to use the biofeedback to lower their arousal at sessions 2 and 3, a technique that the VRET-alone group did not learn, and this would have resulted in the lower self-reported arousal at each pause of the self-guided VRET. People with high FNE have a greater P2 amplitude, an event-related potential, during angry faces relative to neutral faces which denotes heightened early attention to negative facial expressions (90). Biofeedback could reduce arousal from such negative attentional bias. Furthermore, biofeedback produces improvement in anxiety, with effect sizes varying from moderate to large due to variation between studies in number of sessions, age and sample size (91). Thus, greater awareness of arousal during social interactions could help socially anxious individuals to regulate their arousal and report lower arousal. Accordingly, the VRET+biofeedback group showed a marginally higher improvement on LSAS–avoidance of performance at the end of treatment. The findings of the study support the evidence of the benefits of biofeedback as an intervention for anxiety (67) and as an intervention alongside VR therapy (53).

#### 4.1.2 Effect of self-guided VRET on long-term improvement

Regardless of the greater reduction of physiological arousal in the VRET+biofeedback group, the VRET-alone group improved more than the VRET+biofeedback group at one month follow-up on LSAS-Fear of Performance and to a lesser extent LSAS-Fear of Social Situations. Thus, learning to regulate arousal from biofeedback over three self-guided VRET sessions may be insufficient to sustain a long-term improvement in social anxiety. Sustaining attention to the stimulus display of the biofeedback is a challenge of biofeedback training (53). Thus, scaffolding self-guided VRET with biofeedback may have temporary rather than sustained benefits, making participants more reliant on this ongoing feedback to maintain improvement in social anxiety. Simply showing biofeedback is ineffective (Weerdmeester, J. W. et al., 2020). Instead, training in breathing in relaxation during the biofeedback may be more rewarding and retain learning (53, 55). Awareness of physiological arousal through biofeedback may diminish confidence in developing active coping strategies to reduce physiological arousal beyond the VRET session which the VRET-alone group may have developed better. Indeed, heightened introceptive awareness increases social anxiety (55). Biofeedback and neurofeedback as a form of therapy must involve operant learning, such as training in interpreting the feedback and being rewarded for achieving learning goals (91, 92). Gamifying the response to the biofeedback, such as receiving a star rating to successful down-regulation of arousal could reinforce learning (20). Participants in the present may have found down-regulating one’s arousal during the biofeedback without being commended for their success frustrating. The challenge of down-regulating one’s arousal during the biofeedback may have diminished the perceived benefits of the self-guided VRET on PSA and social anxiety. Nonetheless, the sustained improvements following self-guided VRET, regardless of biofeedback, espouse the long-term benefits of the self-guided VRET for social anxiety.

The lived experiences of self-guided VRET may give further insight into the observed effects of the self-guided VRET. Participants provided written feedback about the benefits of the self-guided VRET at one-month follow-up. Participants expressed that the therapy made them more relaxed and less anxious, and it increased their confidence with delivering presentations, even helping some to get a distinction on an assessed presentation,

“I definitely felt more relaxed towards the end of the experiment. It helped me to talk slower and focus on my breathing to relax myself. It also made me aware of certain verbal ticks [sic] that I use when giving a speech.”

Participants also benefitted from the repeated practice even if they felt that the virtual environment could have been more realistic,

“It was very interesting, the environment was very cool, but could be improve upon if slightly more realistic. I’m glad I took part, I think the repetition and having a talk prepared was a really good idea. I think it definitely improved my nervousness.”

### 4.2 Perceived control and risk appraisal as a mechanism for the efficacy of the SGV for social anxiety

Avoidance behaviour as a hallmark of anxiety disorder is facilitated through perceived control over exposure to uncertainty (29). Higher intolerance of uncertainty over future events is linked to greater perceived control over avoidant behaviour (29). Here (29), perceived control was measured as a relief over averting an aversive unconditioned stimulus (a loud noise) after encountering the conditioned stimuli (innocuous images) (29). Such intolerance of uncertainty could perpetuate avoidant behaviour. BIS-appraisal – the tendency to monitor and appraise risk under conditions of uncertainty – underpins elevated trait anxiety and avoidance motivation (27). BIS-appraisal (83) was used a proxy measure of perceived control in this study and it fully explained the improvement in PSA and social anxiety following self-guided VRET. The BIS-appraisal measure denotes the ability to weigh the pros and cons of a situation before engaging with it. This ability to appraise situations could aid appraisal of threat during self-guided VRET, inspire confidence in the benefits of the therapy and reduce avoidant behaviour. Indeed, greater perceived control encourages socially anxious individuals to give better performances (33). Thus, a prior ability to carefully understand situations and stay in control could give anxious individuals more control over their self-guided graded exposure, more confidence in self-guided VRET and improve social anxiety.

### 4.3 Limitations and future research

This study had a high attrition rate by the end, with the percentage of dropout increasing from 10% at session 2 to 55% at follow-up in the VRET+biofeedback group and from 19% at session 2 to 36% at follow-up in the VRET-alone group (**Table 3**). Non-completers were less likely to feel present in the virtual environment. The immersion and realism of the self-guided VRET experience could be improved to address the dropout rate and participant written feedback. Adverse effects were not routinely monitored, but some participants withdrew due to adverse events. Adverse events included distress arising from exposure to the virtual audience, finding the public-speaking challenging, having physical discomfort after the first session and becoming anxious. Participants were not guided about how to lower heartrate during biofeedback which could have undermined confidence in coping strategies and hindered the sustained improvement in social anxiety. We have developed a machine learning algorithm for integrating heartrate, FAA and other cognitive-performance-based measures into multi-sensory integrated feedback (93). Such machine-learning algorithms are based on prolonged, rather than momentary, physiological responses and could prove more reliable to participants. Lastly, asking participants to give a presentation in front of a real audience would help to understand how participants apply learning about arousal from biofeedback in real-world situations. The limited realism of the virtual audience was noted by participants in their written feedback. Encountering animated emotions in the virtual environment may have limited relevance when faced with the social judgements of a real audience. Thus, the fidelity of the intervention must be tested in front of a real audience.

## 5 Conclusion

Three weekly sessions of self-guided VRET produce a clinically meaningful improvement in PSA and social anxiety up to one month after therapy. The accompanied reduction in heart rate reinforces the objective benefits of this self-guided VRET. People with a sub-clinical level of social anxiety could do the self-guided VRET as homework (20) before social situations, such as interacting with family and peers, use of public spaces, job interviews and other performance situations. Furthermore, VRET+biofeedback reduced heartrate and FAA and maintained a steady level of physiological and self-reported arousal. VRET+biofeedback also reduced social avoidance in performance situations marginally more than VRET-alone. The heightened physiological awareness from biofeedback may explain the responsiveness of self-guided VRET since FAA is linked to social withdrawal (47). Being able to examine the virtual environment and focus on the assigned activities was important in improving the experience of the self-guided VRET, since this ability predicted the improvement in PSA. These benefits of the self-guided VRET could help socially anxious individuals who are on a waitlist for treatment from a therapist. These benefits could also help meet the targets of clinical services to offer treatment within six weeks, reduce the burden on clinical services, reduce costs of a trained therapist and reduce therapist burnout that causes errors of judgement (94).

Furthermore, greater perceived control in terms of weighing the pros and cons of a situation before engaging with it explained the improvement in PSA and social anxiety. Teaching participants practical strategies to manage perceived control over impending threat and uncertainty during VRET sessions could sustain the long-term benefits of self-guided VRET (29). Deterioration rates of virtual-reality therapies are comparable with other active therapies (95); this finding alongside the practical benefits of our self-guided VRET increases the credibility of self-guided VRET as a viable accessible therapy to encourage engagement with *in vivo* therapies.

## Data Availability

The raw data presented in the study are publicly available. This data can be found here: https://doi.org/10.5281/zenodo.13995305. Further inquiries can be directed to the corresponding author/s.
