# Corrigendum: Augmenting self-guided virtual-reality exposure therapy for social anxiety with biofeedback: a randomised controlled trial

**DOI:** 10.3389/fpsyt.2025.1548762

**Published:** 2025-02-25

**Authors:** Preethi Premkumar, Nadja Heym, James A. C. Myers, Phoebe Formby, Steven Battersby, Alexander Luke Sumich, David Joseph Brown

**Affiliations:** ^1^ Division of Psychology, London South Bank University, London, United Kingdom; ^2^ Department of Psychology, Nottingham Trent University, Nottingham, United Kingdom; ^3^ Department of Psychology, University of Sheffield, Sheffield, United Kingdom; ^4^ Independent Researcher, Nottingham, United Kingdom; ^5^ Department of Computer Science, Nottingham Trent University, Nottingham, United Kingdom

**Keywords:** social anxiety, longitudinal, perceived control, physiological arousal, presence

In the published article, there were errors in the **Abstract**, *Methods* and *Results*. The number of therapy sessions and position of the subheadings were incorrect. The **Abstract**, *Methods* and *Results* previously stated:

“**Methods**: Seventy-two individuals with high self-reported social anxiety were randomly allocated to VRET-plus-biofeedback (n=38; 25 completers) or VRET alone (n=35; 25 completers). Three hour-long VRET sessions were delivered over two consecutive weeks. During each session, participants delivered a 20-minute public speech in front of a virtual audience.

**Results:** Participants in the VRET-plus-biofeedback group received biofeedback on heartrate and frontal alpha asymmetry (FAA) within the virtual environment and were asked to lower their arousal accordingly. Participants in both groups completed psychometric assessments of social anxiety after each session and at one-month follow-up. PSA improved by the end of treatment and overall social anxiety improved one month after the VRET across both groups. The VRET-plus-biofeedback group showed a steadier reduction in FAA in the first VRET session and a greater reduction in self-reported arousal across the two sessions than the VRET-alone group.”

The corrected version appears below:

“**Methods:** Seventy-two individuals with high self-reported social anxiety were randomly allocated to VRET-plus-biofeedback (n=38; 25 completers) or VRET alone (n=35; 25 completers). Three hour-long VRET sessions were delivered over three consecutive weeks. During each session, participants delivered a 20-minute public speech in front of a virtual audience. Participants in the VRET-plus-biofeedback group received biofeedback on heartrate and frontal alpha asymmetry (FAA) within the virtual environment and were asked to lower their arousal accordingly. Participants in both groups completed psychometric assessments of social anxiety after each session and at one-month follow-up.

**Results:** PSA improved by the end of treatment and overall social anxiety improved one month after the VRET across both groups. The VRET-plus-biofeedback group showed a steadier reduction in FAA in the first VRET session and a greater reduction in self-reported arousal across the three sessions than the VRET-alone group.”

The authors apologize for these errors and state that this does not change the scientific conclusions of the article in any way. The original article has been updated.

